# Supplementary material for: Predictive factors of venous recanalization in upper-extremity vein thrombosis
Source: PLoS One. 2021 May 13;16(5):e0251269. doi: 10.1371/journal.pone.0251269 (PMC8118536; doi:10.1371/journal.pone.0251269)
Supplement: Attachment — Submitted filename: response to reviewers.docx [file pone.0251269.s002.docx]

Dear Editor,

I am writing you the responses to the reviewers. Every comment has been taken into account.

You will find below the details of the answers.

Sincerely

Dr Olivier Espitia

1. Please ensure that your manuscript meets PLOS ONE's style requirements, including those for file naming. The PLOS ONE style templates can be found at

https://journals.plos.org/plosone/s/file?id=wjVg/PLOSOne_formatting_sample_main_body.pdf and

[https://journals.plos.org/plosone/s/file?id=ba62/PLOSOne_formatting_sample_title_authors_affiliations.pdf](about:blank)

Manuscript has been changed, now it respects PLOS ONE style.

2. Thank you for stating the following in the Acknowledgments Section of your manuscript:

'OE received mobility grant, from Société Française de Médecine Vasculaire.'

We note that you have provided funding information that is not currently declared in your Funding Statement. However, funding information should not appear in the Acknowledgments section or other areas of your manuscript. We will only publish funding information present in the Funding Statement section of the online submission form.

a. Please remove any funding-related text from the manuscript and let us know how you would like to update your Funding Statement. Currently, your Funding Statement reads as follows:

'The author(s) received no specific funding for this work.'

b. Please include your amended statements within your cover letter; we will change the online submission form on your behalf.

The changes have been made in the manuscript. Funding information is now in Funding Statement section. The details of these changes were given in the cover letter.

3. We note that you have indicated that data from this study are available upon request. PLOS only allows data to be available upon request if there are legal or ethical restrictions on sharing data publicly. For information on unacceptable data access restrictions, please see http://journals.plos.org/plosone/s/data-availability#loc-unacceptable-data-access-restrictions.

In your revised cover letter, please address the following prompts:

a) If there are ethical or legal restrictions on sharing a de-identified data set, please explain them in detail (e.g., data contain potentially identifying or sensitive patient information) and who has imposed them (e.g., an ethics committee). Please also provide contact information for a data access committee, ethics committee, or other institutional body to which data requests may be sent.

b) If there are no restrictions, please upload the minimal anonymized data set necessary to replicate your study findings as either Supporting Information files or to a stable, public repository and provide us with the relevant URLs, DOIs, or accession numbers. Please see http://www.bmj.com/content/340/bmj.c181.long for guidelines on how to de-identify and prepare clinical data for publication. For a list of acceptable repositories, please see http://journals.plos.org/plosone/s/data-availability#loc-recommended-repositories.

We will update your Data Availability statement on your behalf to reflect the information you provide.

There is no restriction; data were available. All relevant data are within the manuscript and its Supporting Information files.

4. Your ethics statement should only appear in the Methods section of your manuscript. If your ethics statement is written in any section besides the Methods, please delete it from any other section.

Manuscript has been changed; ethics statement should only appear in the Methods section.

5. We noticed you have some minor occurrence of overlapping text with the following previous publication(s), which needs to be addressed:

- https://journals.lww.com/md-journal/Fulltext/2020/02070/A_STROBE_cohort_study_of_755_deep_and_superficial.27.aspx

The text that needs to be addressed involves the Introduction.

In your revision ensure you cite all your sources (including your own works), and quote or rephrase any duplicated text outside the methods section. Further consideration is dependent on these concerns being addressed.

Introduction has been changed.

Additional Editor Comments:
The manuscript is potentially interesting but some revisions are needed.
Reviewers' comments:
Reviewer's Responses to Questions

**Comments to the Author**

1. Is the manuscript technically sound, and do the data support the conclusions?
The manuscript must describe a technically sound piece of scientific research with data that supports the conclusions. Experiments must have been conducted rigorously, with appropriate controls, replication, and sample sizes. The conclusions must be drawn appropriately based on the data presented.

Reviewer #1: Yes

2. Has the statistical analysis been performed appropriately and rigorously?

Reviewer #1: Yes

3. Have the authors made all data underlying the findings in their manuscript fully available?
The [PLOS Data policy](http://antiphishing.aphp.fr/1/b2xpdmllci5lc3BpdGlhLWV4dEBhcGhwLmZyfFZSQzEzNzAzMjk%3D/www.plosone.org/static/policies.action%23sharing) requires authors to make all data underlying the findings described in their manuscript fully available without restriction, with rare exception (please refer to the Data Availability Statement in the manuscript PDF file). The data should be provided as part of the manuscript or its supporting information, or deposited to a public repository. For example, in addition to summary statistics, the data points behind means, medians and variance measures should be available. If there are restrictions on publicly sharing data—e.g. participant privacy or use of data from a third party—those must be specified.

Reviewer #1: Yes

4. Is the manuscript presented in an intelligible fashion and written in standard English?
PLOS ONE does not copyedit accepted manuscripts, so the language in submitted articles must be clear, correct, and unambiguous. Any typographical or grammatical errors should be corrected at revision, so please note any specific errors here.

Reviewer #1: No

Manuscript has been changed and native English speaker reviewed the manuscript.

5. Review Comments to the Author
Please use the space provided to explain your answers to the questions above. You may also include additional comments for the author, including concerns about dual publication, research ethics, or publication ethics. (Please upload your review as an attachment if it exceeds 20,000 characters)

Reviewer #1: I have a number of questions regarding this manuscript:

1. In the abstract, in line 47, occlusive thrombus is associated with complete recanalization (HR 0.48). Then in the conclusion, it states that the “absence of occlusive thrombus” as one of the factors associated with UEVT recanalization. I understand since the HR is 0.48 that the negative of occlusive thrombus is the factor associated with complete recanalization, but I must say it is initially confusing to have it say “occlusive thrombus” in the Results and then the “absence of occlusive thrombosis” in the Conclusion. Can the authors please be consistent in their wording?
Abstract has been changed: Occlusive thrombosis was associated with the absence of UEVT recanalization.

2. In the Abstract in line 44, what is the meaning of the term “presence of endovascular material in 86.4%”? Is this scar tissue within the vein from a previous DVT? Is so, then the authors should state this rather than the non-descript terminology they use.

Endovascular material referred to venous catheter or pacemaker leads. Changes have been made in the manuscript.

3. On page 4, line 68, when is kidney failure non-serious? I would remove the word serious in front of kidney failure, or change to….kidney failure requiring dialysis.

Serious has been deleted and replaced by acute kidney failure or kidney failure requiring dialysis.

4. On page 5, line 87, what does the term …” adherent thrombus whose major axis was >5 mm” mean?
We have clarified the definition of venous blood clot and modified the sentence: UEVT linked to the CVC was defined by a blood clot facing the catheter with blood clot in contact with the vein wall with a length > 5 mm

5. Can the authors be more complete in the description of the two patients who died “related to major bleedings under anticoagulant therapy” on page 8, lines 158-159? What anticoagulant were they on, where was the bleeding, etc.? This is an important piece of information that they should share with the readers.

Indeed, it’s interesting data. These two patients are now described in the manuscript. They had therapeutic LMWH, none had over dosage.

“Two deaths were considered to be related to UEVT (0.4%): none related to PE and 2 related to major bleedings under anticoagulant therapy. A 73-year-old man with gallbladder cancer treated with therapeutic LMWH who have gastrointestinal bleeding and a 49-year-old woman postoperatively after cardiac surgery with ECMO under therapeutic LMWH who have a fatal intracerebral hemorrhage. None had LMWH over dosage.”

6. I was surprised on page 9, line 171-172 that the median treatment time was the same for patients with UEVT and a central catheter who had both catheter continuation and catheter removal. I would expect that with catheter continuation, anticoagulation would have remained intact and not stopped, while with catheter removal, anticoagulation could more readily stop. Can the authors please explain this?

I agree with you about the duration of anticoagulation which was the same depending on whether the catheter was removed or not.

However, there are several factors that explain this lack of difference. On the one hand, the duration of anticoagulation treatment was not standardized and depended on the practice of each physician taking care of these patients. On the other hand, many of these patients had active neoplasia at the time of thrombosis, and the anticoagulant treatments were therefore prolonged because of active treatment of the cancer, whether the catheter was removed or not.

Changes have been made in the manuscript in limits section page 13 line 247.

7. I would have expected that an important finding from this study is that the rate of recanalization without squeal of UEVT was low (38%) with a median time to recanalization of 43 days, as reported in the first sentence of the Conclusion. Why is this not included in the abstract? Why is this fact buried in the middle of the Results (page 9, line 175)?

I thank the reviewer for this remark, I fully agree. We have put forward this data which is now quoted in the abstract and in the first part of the conclusion.

8. Finally, I am concerned about mixing superficial thrombosis in with DVT in this study? Not only are they different entities with different treatment modalities, they are called by different names in the paper…..” superficial thrombosis of the forearm” in the Abstract and “superficial forearm UEVT” in the Conclusion. This is confusing on multiple levels. Would the authors consider moving superficial thrombosis out of the paper, or separating this away from DVT?

We understand the comment made by the reviewer, but this study analyzed all the venous thromboses of the upper limbs, which have never been reported before. It analyzed numerous venous territories: deep and proximal, deep and distal, superficial of the arm and forearm.

However, we agree with the reviewer's opinion on proximal venous thrombosis because of a daily clinical problem. Thus, we performed in 304 patients’ new analyses on the factors associated with venous recanalization studying only deep and proximal venous thrombosis with cox model.

We performed a univariate analysis presented in Table 3 and then a multivariate analysis with a stepwise selection. We started with 6 variables: presence of a central venous catheter, infectious context, number of thrombosed venous segments, context of neoplasia, non-occlusive thrombosis, and sex. The final analysis is presented in Table 3. The presence of a central venous catheter, infectious context, and non-occlusive thrombosis were factors associated with venous recanalization.

I our opinion, data on distal thromboses are also interesting; as there is very little data on superficial vein thrombosis of the upper limbs, we wish to present these data in the manuscript, to better understand the factors associated with their recanalization. Thus, in time, their treatments could be better codified.

It is important to differentiate distal veins of the arm, particularly the basilic veins and the humeral veins, which are frequently used for PICC Line placement, and superficial veins of the forearm which are very often punctured for blood sampling.

We have clarified these notions in the manuscript. When analyzing the factors associated with recanalization, it was only superficial forearm venous thrombosis and not superficial arm venous thrombosis.

Changes have been made in the manuscript and superficial thrombosis of the forearm is now the only formulation used in the manuscript.
