# Supplementary material for: Predictive factors of venous recanalization in upper-extremity vein thrombosis
Source: PLoS One. 2021 May 13;16(5):e0251269. doi: 10.1371/journal.pone.0251269 (PMC8118536; doi:10.1371/journal.pone.0251269)
Supplement: Attachment — Submitted filename: R2 response to reviewers.docx [file pone.0251269.s003.docx]

Dear Editor,

Please find enclosed a revised manuscript entitled “**Predictive factors of venous recanalization in upper-extremity vein thrombosis**” for submission to your journal.

All the remarks were taken into account. The answers are detailed in the response to the reviewer file.

Sincerely

Dr Olivier Espitia

Review Comments to the Author

Please use the space provided to explain your answers to the questions above. You may also include additional comments for the author, including concerns about dual publication, research ethics, or publication ethics. (Please upload your review as an attachment if it exceeds 20,000 characters)

Reviewer #1: I have two additional comments for the authors.

1. On page 9, line 167 in the revised final version, please change "None had LMWH over dosage" to "Neither of these two patients had an over dosage of LMWH".

The changes have been made in the manuscript.

2. On page 13, line 248 in the revised final version, please change the abbreviation "PM" to what it actually means.

PM means pace-maker, the abbreviation has been changed in the manuscript.
